# A pilot proof-of-concept study of microbial and botanical diversity in honey samples from Necochea, Argentina

**DOI:** 10.3389/fmicb.2026.1833002

**Published:** 2026-05-14

**Authors:** R. Taussig, R. Peralta, J. P. Bustamante

**Affiliations:** 1 Departamento de Ingeniería Biomédica, Facultad de Ingeniería, Universidad Austral, Pilar, Argentina; 2 Facultad de Ingeniería, Universidad Nacional de Entre Ríos, Oro Verde, Argentina; 3 Centro Interdisciplinario de Investigaciones en Ciencias de la Salud y del Comportamiento (CIICSAC, CONICET-UAP), Facultad de Ciencias de la Salud, Universidad Adventista del Plata, Libertador San Martin, Entre Ríos, Argentina

**Keywords:** apiculture, botanical diversity, honey, metagenomics, microbial diversity, shotgun sequencing

## Abstract

**Introduction:**

Honey is a complex biological matrix containing plant-derived, microbial, and viral components that reflect both environmental and hive-associated processes. Traditional methods for determining botanical origin, such as melissopalynology, have limitations in resolution and scope. In this context, untargeted shotgun metagenomics emerges as a promising integrative approach for comprehensive honey characterization.

**Methods:**

This pilot study explored the feasibility of applying an untargeted shotgun metagenomic approach to honey samples from Necochea, Buenos Aires province, Argentina. Two honey samples and a pollen control sample from Rosa chinensis were subjected to DNA extraction, shotgun library preparation, and sequencing on an Illumina NextSeq 500 platform.

**Results:**

The control sample showed exclusive assignment to *Rosa chinensis*, supporting the validity of the analytical workflow. In both honey samples, plant-derived sequences were predominantly assigned to *Helianthus annuus* (common sunflower) and *Eucalyptus grandis* (rose gum), consistent with the regional flora. Key bacterial taxa included *Paenibacillus larvae* in one sample, *Acinetobacter johnsonii* in the other, and *Apilactobacillus kunkeei, Bradyrhizobium* sp., *Sphingobium yanoikuyae*, and *Stutzerimonas stutzeri* in both. *Apis mellifera filamentous virus* was detected in both samples.

**Discussion:**

Given the limited sample size, these findings should be interpreted as exploratory and hypothesis-generating. Nevertheless, this proof-of-concept supports the potential of untargeted metagenomics as an integrated tool for the simultaneous characterization of botanical origin, microbial communities, and viral content in honey, offering advantages over targeted amplicon-based approaches. Future studies with larger and systematically designed cohorts will be necessary to validate and extend these observations.

## 1 Introduction

Honey is a widely consumed natural product, valued primarily for its nutritional properties and health benefits. It is well-known as a sweet substance produced by bees from plant nectar, secretions from living plant tissues, or insect excretions. Bees collect and transform these raw materials by combining them with specific endogenous secretions. Because bees forage across a wide variety of plant species, honey can originate from a broad range of botanical sources. Botanical origin is a key quality attribute and an important factor in consumer confidence. Honey can have different commercial values depending on its floral source. Based on botanical origin, honey is classified as monofloral (unifloral) or multifloral (polyfloral), depending on whether it derives predominantly from one or multiple plant species, respectively (Balkanska et al., 2020). In general, honey is an extremely complex matrix. Its composition depends on the floral origin of the nectar and the plant species visited by the bees. Sugars are its principal components, with fructose and glucose accounting for approximately 65% of the total sugars as the dominant monosaccharides. Honey also contains minor components such as proteins, enzymes, amino acids, organic acids, vitamins, volatile compounds, phenolic acids, and flavonoids (Ouchemoukh et al., 2007; Silva et al., 2009; Wu et al., 2017).

The botanical identification of honey can be achieved through analysis of its various components, relying on melisopalynological, chemical (amino acid and protein profiling, sugar analysis, aromatic compounds, mineral elements), and genetic-material-based techniques (Balkanska et al., 2020). In this study, we seek to identify the botanical diversity of honey samples through extraction and sequencing of the genetic material present in the samples via metagenomic analysis. The advantage of this technique lies in its ability to leverage the full bioinformatic toolkit developed by our research group, while expanding the reference database to include the plant kingdom. Additionally, this approach offers the added benefit of enabling simultaneous microbial profiling of each sample.

## 2 Materials and methods

### 2.1 Sample origin

Two honey samples of commercial origin were obtained from Necochea (Buenos Aires Province, Argentina). No formal or randomized design was implemented. Instead, samples were obtained from a commercial origin with the specific intention of conducting an exploratory analysis. The rationale was to test whether shotgun metagenomics could recover meaningful biological signals from honey matrices under real-world, non-controlled conditions. In order to establish a reference and validate the lab procedures employed, a control sample was prepared using pollen of known botanical origin -rose from a residential garden, *Rosa chinensis*- and processed in the same batch together with the honey samples. The choice of *R. chinensis* is justified by its membership in the *Rosaceae* family, one of the most frequent pollen types in honey palynological spectra (Herrero et al., 2002), and by its local availability as a well-characterized reference plant.

Botanical origin of honey was not determined using conventional melissopalynological approaches (i.e., pollen identification by light microscopy). The study focused exclusively on the genetic determination of the origin of the pollen present in the samples.

### 2.2 DNA extraction and sequencing

One of the main challenges in this work was obtaining high-purity genetic material from honey samples, given the complex composition of honey, its viscous texture, and the difficulties associated with handling it on the lab bench. The lab protocol consisted of a honey pretreatment step to extract pollen grains or any other cells from which genetic material could be obtained. Fifty grams of honey were distributed across 4 × 50 mL tubes (12.5 g honey per tube), filled to volume with molecular biology grade water, free of DNases and RNases. Samples were incubated at 40 °C for 1 min and then centrifuged at 5,000 × *g* for 25 min. After discarding the supernatant, 5 mL of distilled water was added to each pellet; the four pellets were then pooled into a single tube and centrifuged again at 5,000 × *g* for 25 min. The resulting pellet was resuspended in 0.5 mL of distilled water and transferred to a 1.5 mL microcentrifuge tube for DNA extraction (Bovo et al., 2020). DNA extraction was subsequently performed following an SDS-based protocol (Xia et al., 2019). DNA concentration was measured with a Qubit® Fluorometer 4, and DNA integrity was assessed by agarose gel electrophoresis to verify the absence of degradation. No primers were used for isolation. Total DNA extraction was performed. All three samples, including the control, were processed under identical laboratory conditions after the DNA extraction, within the same batch to ensure technical consistency. Although the DNA concentrations obtained after extraction were low (Honey A: 0.247 ng/μL; Honey B: 0.129 ng/μL; Control: 0.235 ng/μL), authors proceeded with shotgun library preparation. This decision was based on both the specifications of the library preparation protocol, which supports low DNA input amounts (including inputs below 100 ng, with appropriate adjustment of amplification cycles), and prior experience of the research team indicating that reliable genomic libraries can be successfully generated even from low-input samples. Consistent with this, post-library preparation concentrations increased substantially (Honey A: 33.77 ng/μL; Honey B: 35.57 ng/μL; Control: 29.50 ng/μL), falling within the expected range for sequencing-ready libraries, thus supporting the adequacy of the workflow. Libraries were prepared from the extracted DNA following the Illumina® DNA Prep protocol, and library concentration was measured using a Qubit® Fluorometer 4. During the library preparation E07, E08 and E09 (IDT-Ilmn DNA–RNA UD Indexes SetA Tagmentation) index adapters were used (Honey A UDP0053F GGAATTGTAA and UDP0053R AGGATGTGCT; Honey B UDP0061F TGGCTAATCA and UDP0061R CTACATGCCT; Control UDP0069F TGGAGTACTT and UDP0069R TCCACACAGA). Library pool quality was assessed on an Agilent Bioanalyzer 2,100. Sequencing was performed on an Illumina® NextSeq500 instrument.

To minimize contamination, all sample handling and DNA extraction procedures were performed following good laboratory practices, including sterile conditions, single-use consumables, DNA-free reagents, physically separated work areas for pre- and post-extraction steps, and routine decontamination of surfaces and equipment. In addition, all samples were processed within the same batch to ensure procedural consistency and reduce the risk of cross-contamination.

### 2.3 Bioinformatic analysis

Raw FASTQ reads were processed using the KneadData pipeline (v0.12.4)^1^ for quality control. Adapter trimming and quality filtering were performed using Trimmomatic (integrated). Removal of human remains contaminants from sample handling in the laboratory was achieved by aligning the reads against the human reference genome (GRCh39/hg39)^2^ using Bowtie2 (integrated).

Taxonomic profiling, for both botanical and microbial classifications, was carried out using Kraken2 (v2.17.1) (Wood et al., 2019) with the *PlusPFP-16* database offered by the tool’s authors and available at the following link: https://genome-idx.s3.amazonaws.com/kraken/k2_pluspfp_16_GB_20251015.tar.gz at the default confidence score threshold (−confidence 0.0), consistent with the software developers’ recommended practice for environmental metagenomics (Wood et al., 2019) (Kraken2 FAQ). Downstream analyses were restricted to direct species-level read counts (rank “S,” direct reads >0).

After obtaining the abundances of each sample, alpha diversity was calculated using the metrics of Shannon, Berger–Parker, Fisher and Simpson implemented in Krakentools (v1.2.1) (Lu et al., 2022).

Comparative analysis between honey samples was performed using three complementary approaches. Beta diversity was quantified using the Bray–Curtis dissimilarity index and the Jaccard similarity index, both calculated from species-level direct read counts obtained from Kraken2 output files. The Bray–Curtis index accounts for differences in relative abundance, while Jaccard reflects qualitative species overlap (presence/absence). To assess the concordance in relative abundances of shared taxa, Spearman’s rank correlation (*ρ*) was calculated across all species detected in both samples simultaneously (*n* = 4,122 shared species), using log_10_-transformed relative abundances. All statistical analyses were performed in Python 3 using NumPy v2.4.0 and SciPy v1.17.1.

**Figure 1:**
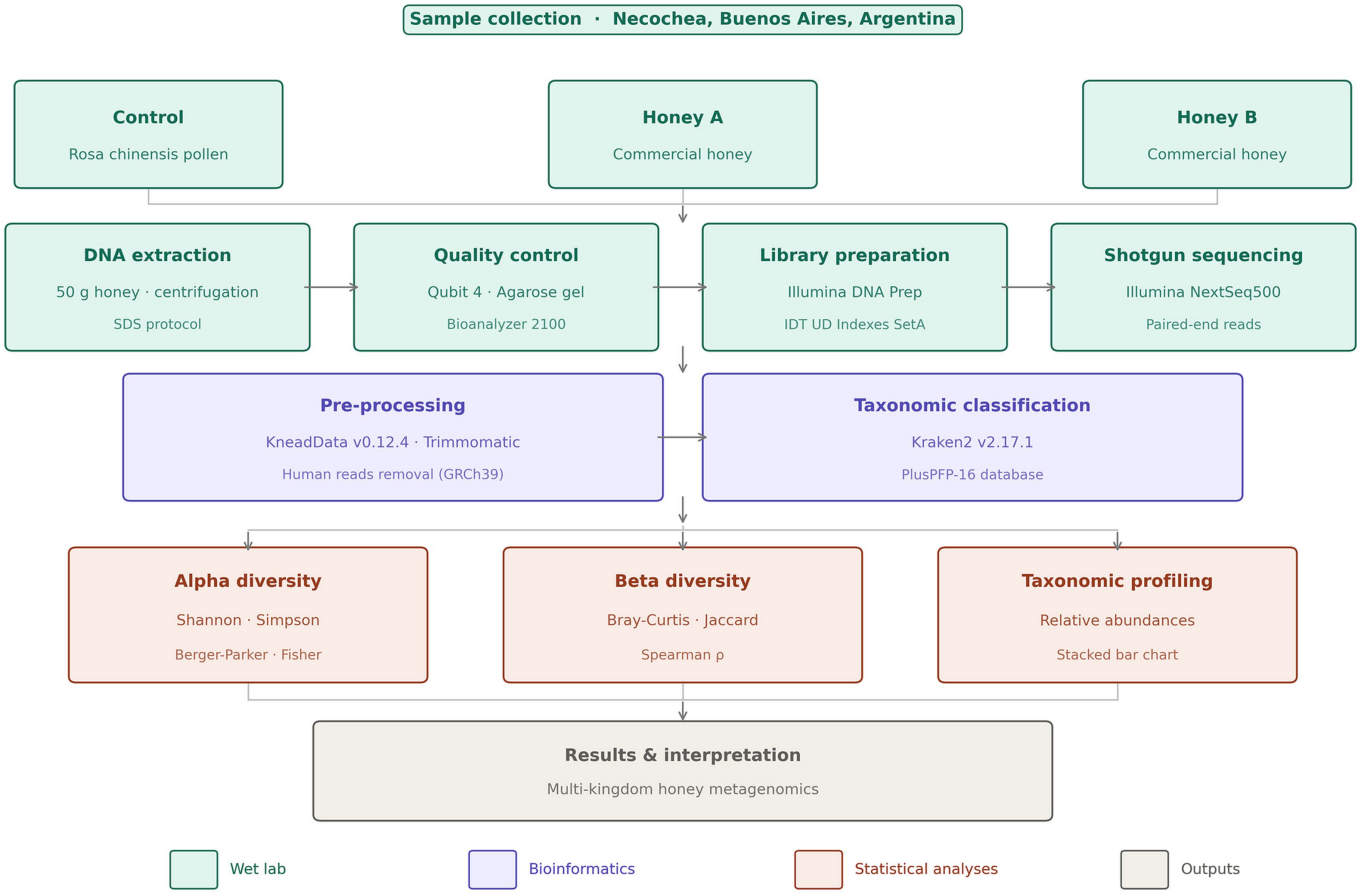
A schematic workflow diagram summarizing the complete methodological pipeline.

A schematic workflow diagram summarizing the complete methodological pipeline, from sample acquisition to bioinformatic analysis and interpretations can be observed at Figure 1.

## 3 Results

Of the total sequencing reads (9,489,653 to Control, 8,698,817 to Honey A and 10,158,383 to Honey B), 61% of those from the control sample were assigned to known taxa in the reference database, compared to 19.5% for honey sample A (Honey A) and 40% for honey sample B (Honey B) (see Table 1).

**Table 1:** Summary of Kraken2 classification results for all samples.

Taxonomic level	Control			Honey A			Honey B		
	Reads	(%)	Taxa	Reads	(%)	Taxa	Reads	(%)	Taxa
Unclassified	3,616,187	38.11%	—	7,003,949	80.52%	—	6,086,198	59.91%	—
Root	34,944	0.37%	3	10,363	0.12%	3	52,527	0.52%	3
Super kingdom	8,641	0.09%	4	10,342	0.12%	6	14,734	0.15%	7
Kingdom	19	0.00%	2	53	0.00%	2	44	0.00%	2
Phylum	2,808	0.03%	9	4,143	0.05%	10	17,611	0.17%	14
Class	143,256	1.51%	21	22,618	0.26%	32	57,244	0.56%	26
Order	7,513	0.08%	36	4,371	0.05%	46	5,197	0.05%	43
Family	136,986	1.44%	101	18,778	0.22%	148	24,535	0.24%	141
Genus	26,376	0.28%	427	61,862	0.71%	642	1,911,198	18.81%	559
Species	5,512,923	58.09%	4,541	1,562,338	17.96%	6,721	1,989,095	19.58%	5,959
Total reads	9,489,653	100.00%	—	8,698,817	100.00%	—	10,158,383	100.00%	—

Among the taxonomically assigned reads, the control sample showed 97.31% classification within the kingdom *Plantae*, specifically *R. chinensis*, consistent with the pollen used as reference material. In Honey A, 58% of the assigned reads mapped to *Plantae*, 10.5% to Bacteria, and 31.5% to viruses. By the way, sample Honey B showed 62.5% of assigned reads mapping to Bacteria, 20% to *Plantae*, and 17.5% to viruses.

**Figure 2:**
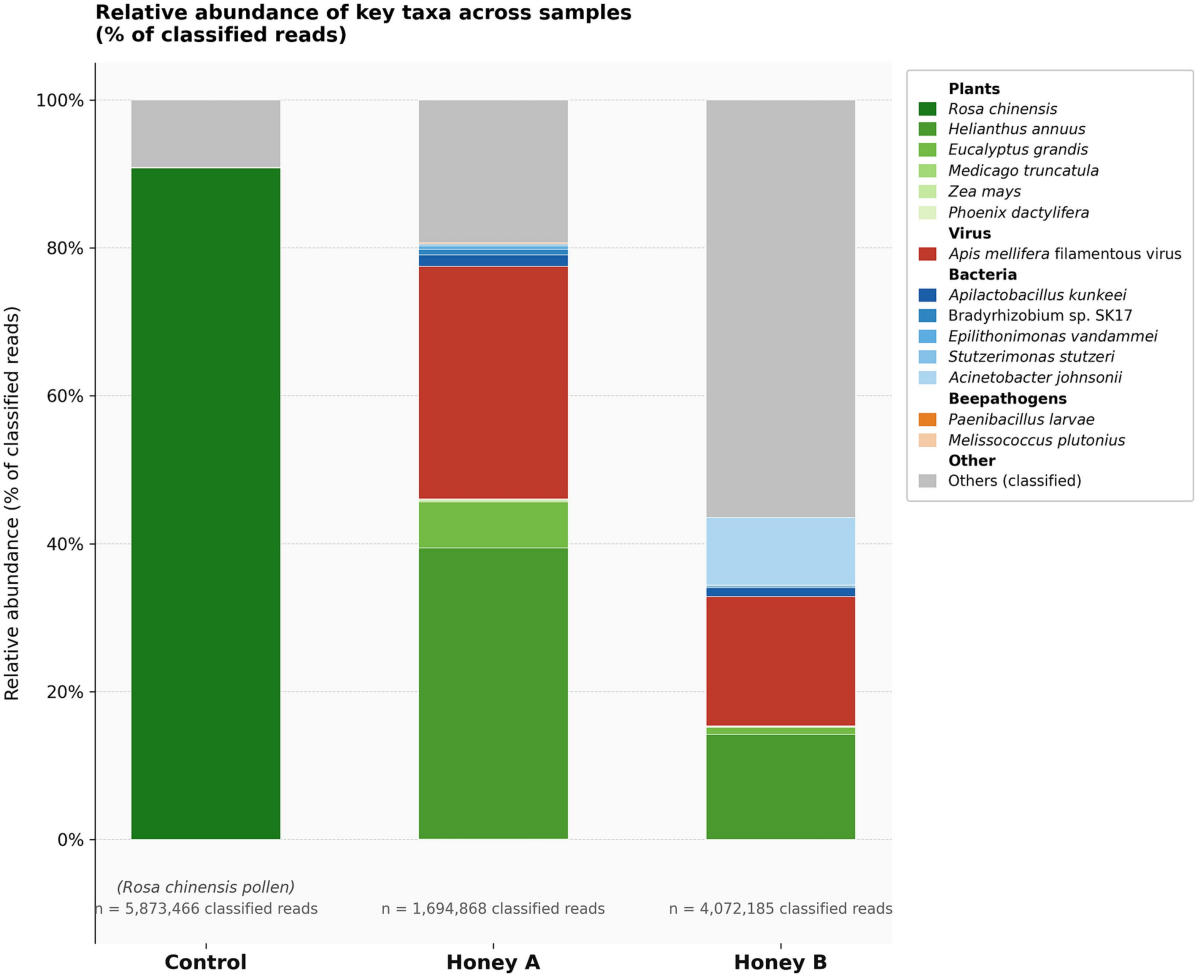
Relative abundance of the main organisms detected in each sample.

Within the plant kingdom, both honey samples were dominated by *Helianthus annuus* (common sunflower) (Badouin et al., 2017), at 70% and 80% of plant-assigned reads for Honey A and Honey B, respectively, followed by *Eucalyptus grandis* (rose gum) (Myburg et al., 2014) detected at lower yet clearly appreciable relative abundance. Among bacterial taxa, notable findings included the detection of *Paenibacillus larvae* in sample Honey A; *Acinetobacter johnsonii, A. lwoffii*, and *A. baumannii* in sample Honey B; and *Apilactobacillus kunkeei*, *Bradyrhizobium* sp., *Sphingobium yanoikuyae*, and *Stutzerimonas stutzeri* in both samples. The only detected viral species in both samples was AmFV (Figure 2). AmFV was detected in both honey samples with substantial read support. In Honey A, 532,395 reads were assigned to AmFV (6.12% of total reads), corresponding to an estimated genome coverage of ~160x. In Honey B, 712,058 reads were assigned (7.01% of total reads), with an estimated coverage of ~214x. Coverage was calculated as (number of reads × 150 bp) divided by the AmFV reference genome length (498,059 bp; NCBI accession GCF_001308775.1). These values indicate a robust and consistent viral signal across both samples.

The alpha diversity values obtained for each of the honey samples were as follows: Species richness (Honey A: 5619; Honey B: 5019), Shannon (Honey A: 1.8966; Honey B: 2.0025), Simpson (Honey A: 0.6890; Honey B: 0.7461), Berger-Parker (Honey A: 0.4313; Honey B: 0.3614), and Fisher (Honey A: 734.05; Honey B: 622.71). The dominant species in sample Honey A was *Helianthus annuus*, and the dominant species in sample Honey B was AmFV.

**Figure 3:**
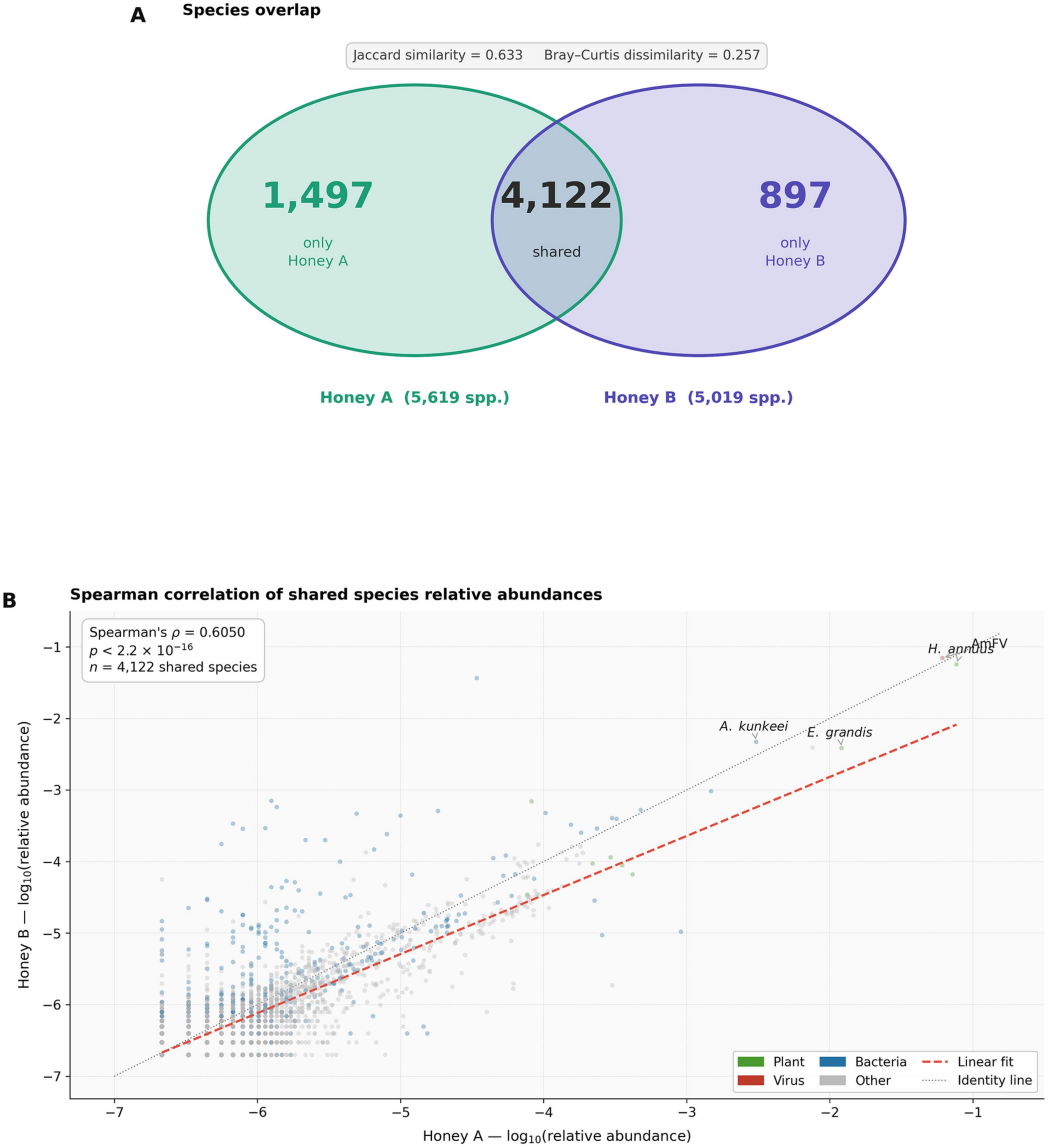
Comparative metagenomic analysis of two honey samples (Honey A and Honey B). **(A)** Venn diagram showing the overlap of species-level taxa detected between both samples. Numbers indicate species exclusive to each sample or shared between them. The Jaccard similarity index (0.633) and Bray–Curtis dissimilarity index (0.257) are shown above the diagram. **(B)** Spearman’s rank correlation of log_10_-transformed relative abundances across the 4,122 species shared between both samples (*ρ* = 0.605, *p* < 2.2 × 10^−16^). Each point represents one species, colored by taxonomic category. The dashed red line indicates the linear regression fit; the dotted gray line indicates the identity line (*y* = *x*). Key species are annotated.

Comparative analysis of the two honey metagenomes revealed substantial community overlap alongside notable compositional differences (Figure 3). The Bray–Curtis dissimilarity between Honey A and Honey B was 0.257, indicating an overall quantitative similarity of 74.3%. The Jaccard similarity index was 0.633, with 4,122 species shared out of a combined pool of 6,516 (63.3%). Despite this overlap, the dominant species differed markedly between samples: *Helianthus annuus* was the most abundant species in Honey A (7.68% of total reads), while AmFV was the dominant species in Honey B (7.01%). Spearman’s rank correlation of log-transformed relative abundances across shared species yielded *ρ* = 0.605 (*p* < 2.2 × 10^−16^, *n* = 4,122), indicating a moderate-to-strong concordance in rank-order abundances between both samples.

## 4 Discussion

In exploratory studies of this nature, the inclusion of a well-characterized reference sample is essential to appropriately contextualize and interpret the findings. By incorporating a pollen sample derived from a taxonomically identified plant species as a control for both the lab procedures and downstream bioinformatic analyses, we establish a benchmark against which the recovered and sequenced genetic material could be evaluated. The complete concordance between the expected and observed taxonomic classification in the control sample provides internal validation of the extraction, library preparation, sequencing, and analytical workflows.

This approach not only strengthens confidence in the lab procedures applied to the honey samples but also supports the robustness and reliability of the bioinformatic pipeline used for taxonomic assignment.

We observed that the most common molecular approach for honey characterization relies on targeted amplicon sequencing of specific genetic markers. These markers are typically used to identify botanical origin (e.g., ITS, rbcL, matK, trnL, psbA-trnH, and COI) (Balkanska et al., 2020; Schnell et al., 2010; Lalhmangaihi et al., 2014; Bruni et al., 2015; Hawkins et al., 2015; Prosser and Hebert, 2017; Oliver et al., 2021; Wirta et al., 2021); determine pollinator bee species under COI, 16S rRNA, or ITS2 (Schnell et al., 2010; Prosser and Hebert, 2017); or detect beneficial or pathogenic bacteria (under 16S rRNA) (Wirta et al., 2021; Olivieri et al., 2012). The main advantage of this targeted strategy lies in its low cost and its long-standing use in the field. However, its inherent limitation is that results are restricted to the specific molecular markers chosen for amplification, discarding all other genetic information present in the sample. In this sense, metagenomic approaches offer a substantially broader analytical scope. Within a single experiment, they enable simultaneous characterization of the botanical origin, identification of bacteria, fungi, and viruses, providing a more comprehensive ecological snapshot of the honey matrix.

In the metagenomic analysis of the control and both honey samples, it is important to note that, on average, approximately 60% of total reads were not assigned to any organism in the reference database. This proportion is consistent with previously reported studies (Bovo et al., 2020). This phenomenon reflects a combination of limitations in reference database completeness, and technical artifacts inherent to sequencing and assembly processes.

Among the successfully assigned reads to organisms present in the reference database, the control sample yielded 97.31% assignment to *R. chinensis*, which was the expected outcome. Within the plant kingdom, the dominant species identified in the honey samples were *Helianthus annuus* (common sunflower) and *Eucalyptus grandis* (rose gum), both widely distributed in the Necochea region from which the samples were collected, reinforcing the ecological plausibility of the findings.

From a bacteriological perspective, the most prominent organism in sample Honey A was *Paenibacillus larvae*, a Gram-positive spore-forming bacterium and the causative agent of American foulbrood (AFB), the most devastating bacterial disease of the honey bee, which infects and decimates larval populations within the hive (Tsourkas, 2020). The detection of *P. larvae* in Honey A warrants careful interpretation given its biological and epidemiological relevance. It is important to note that shotgun metagenomic sequencing detects DNA and does not provide direct evidence of microbial viability or infectivity. Therefore, the presence of *P. larvae* sequences in this sample should be interpreted as indicative of the presence of genetic material, which may derive from viable cells, dormant spores, or residual environmental DNA. This distinction is particularly relevant in the context of spore-forming bacteria such as *P. larvae*, whose spores are highly resistant and can persist in honey and hive-related materials for extended periods (Tsourkas, 2020). Consequently, the detection of *P. larvae* DNA in honey does not necessarily reflect active infection within a colony but may instead represent environmental contamination or historical exposure. Additionally, the possibility of false positives or misclassification inherent to metagenomic approaches should be considered. However, in this case, *P. larvae* was detected at a relative abundance of 0.434%, which reduces the likelihood that this signal arises solely from background noise or spurious low-level assignments. Importantly, no targeted validation (e.g., quantitative PCR or culture-based assays) was performed in this study to confirm the presence or viability of *P. larvae*. As such, this finding should be regarded as preliminary and hypothesis-generating. Future studies incorporating targeted detection methods, viability assays, and longitudinal sampling of hives will be essential to determine the biological and epidemiological significance of these observations.

Looking at sample Honey B, the dominant bacterium was *Acinetobacter johnsonii*; together with *A. lwoffii,* this species belongs to a group of *Acinetobacter* taxa predominantly found in environmental settings (Carvalho et al., 2024). Both samples showed detection of *Apilactobacillus kunkeei*, a fructophilic lactic acid bacterium found in fructose-rich environments such as flowers, fruits, fermented foods, honey, and molasses, as well as in the gut of fructose-feeding insects (Endo and Salminen, 2013); *Bradyrhizobium* sp., associated with oilseed crops and involved in soil nitrogen fixation (Stackebrandt, 2001); *Sphingobium yanoikuyae*, which plays a role in bioremediation and biodegradation of pollutants in sediments and sandy soils (Jou et al., 2022); and *Stutzerimonas stutzeri,* which performs important ecological functions including nitrogen fixation and contaminant degradation (Ge et al., 2023). From a virological standpoint, AmFV was detected in both honey samples. Although AmFV can be pathogenic under acute or stress-associated conditions, it is frequently reported as endemic in honey bee colonies and often present without overt clinical manifestations (Papp et al., 2024). Its detection, therefore, may represent background viral carriage rather than active disease, underscoring the importance of contextual interpretation in metagenomics datasets.

Collectively, these microorganisms reflect complex ecological interactions linking plant communities, environmental reservoirs, and hive-associated microbiota. The estimated genome coverage reported for AmFV should be interpreted as an approximation derived from taxonomic assignment (using Kraken2) and assumes a uniform distribution of reads across the reference genome. A more accurate assessment of genome coverage would require direct read mapping against the reference genome using alignment-based approaches such as Bowtie2 or BWA, which was beyond the scope of the present study. From a biological perspective, although AmFV has been associated with pathogenic effects under certain stress-related or acute conditions, it is also widely recognized as an endemic virus in honey bee populations and is frequently detected in the absence of overt clinical manifestations. Therefore, despite the strong and consistent detection observed in both samples, its presence should be interpreted within this ecological context and does not necessarily indicate active infection or disease. Rather, it likely reflects background viral carriage within bee populations contributing to the honey matrix.

Both honey samples exhibited moderate overall alpha diversity values, with Shannon indices of 1.90 (Honey A) and 2.00 (Honey B), within the range typically reported in metagenomic studies of honey and bee-associated environments (Bovo et al., 2018; Galanis et al., 2022). The Berger–Parker dominance index revealed that Honey A was strongly dominated by *Helianthus annuus* (*d* = 0.43), reflecting a predominantly sunflower botanical origin, while in Honey B the dominant species was AmFV (*d* = 0.36). The detection of AmFV at high genome coverage (160x and 214x in Honey A and Honey B, respectively) is consistent with previous shotgun metagenomic studies of honey that reported near-complete AmFV genome coverage in both monofloral and polyfloral samples (Bovo et al., 2018). Despite high species richness (S = 5,619 and S = 5,019, respectively), the low Shannon values reflect the uneven distribution characteristic of honey metagenomes, where a few taxa account for the vast majority of reads. The elevated proportion of unclassified reads (80.52% in Honey A and 59.91% in Honey B) is consistent with the findings of Bovo et al. (2020), who reported that only ~5% of reads from polyfloral honey samples could be annotated against the NCBI nt database, highlighting the underrepresentation of honey-associated taxa in current reference databases. The botanical origin of the honey samples was a determining factor in shaping community composition, in agreement with Xiong et al. (2023), who demonstrated that honey type is a significant driver of alpha and beta diversity metrics across bacterial and fungal communities in raw honey.

The moderate Bray–Curtis dissimilarity (0.257) and high Jaccard similarity (0.633) observed between the two honey samples suggest that, despite originating from different colonies or foraging contexts, both metagenomes share a substantial core of detectable taxa. This is consistent with findings from Bovo et al. (2018), who reported that honey metagenomes capture a reproducible multi-kingdom signature reflecting both the hive ecosystem and the surrounding flora, and with Galanis et al. (2022), who demonstrated that honey shotgun metagenomics produces community profiles consistent across seasonal samplings. The moderate Spearman correlation (*ρ* = 0.605) further indicates that while both samples track similar ecological sources, quantitative differences in dominant species—particularly the shift from plant-dominated (Honey A) to virus-dominated (Honey B) reads—reflect relevant biological variation between colonies that warrants further investigation, especially in the context of AmFV prevalence and its potential impact on colony health (Bovo et al., 2018; Galanis et al., 2022).

A single positive control consisting of pollen from a known botanical origin (*R. chinensis*) was included to validate the overall analytical workflow, from DNA extraction to taxonomic assignment. This control confirmed the ability of the pipeline to accurately recover botanical signals under the same experimental conditions as the honey samples. However, no negative extraction controls (e.g., blank samples) were included, which represents a limitation of the study and restricts the ability to fully assess background contamination. Potential sources of contamination should therefore be considered when interpreting the results. Laboratory-derived contamination (e.g., from reagents or consumables) was minimized through the use of sterile, single-use materials, DNA-free reagents, physically separated work areas for pre- and post-extraction steps, and batch processing of all samples. Nonetheless, low-level contamination cannot be entirely excluded. Environmental DNA is also expected to contribute to the complexity of honey matrices, potentially influencing the detection of low-abundance taxa. In addition, the possibility of index hopping associated with sequencing on the Illumina NextSeq 500 platform should be acknowledged, as it may introduce low-level cross-sample signal. However, given the relatively high abundance of several key taxa and viral sequences reported in this study, it is unlikely that such technical artifacts alone account for the observed patterns. Future studies incorporating dedicated contaminant-filtering strategies will be essential to further strengthen the robustness of metagenomic analyses in this context.

A key limitation of this study is the restricted sample size, which precludes broad generalization of the findings. While larger and more systematically designed datasets would be necessary to draw robust conclusions, such expansion was not feasible within the scope of this work due to logistical and budgetary constraints, as well as the discontinuation of the original project framework.

Accordingly, the results presented here should be interpreted as exploratory and hypothesis-generating. Future studies incorporating larger sample sizes, controlled sampling strategies, and biological replication will be essential to validate these observations and to further assess the applicability of shotgun metagenomics for comprehensive honey characterization.

The use of untargeted shotgun metagenomics in this study highlights several advantages over conventional metabarcoding approaches. Unlike targeted amplicon-based methods, which rely on predefined primer sets and are therefore subject to amplification bias and limited taxonomic resolution, shotgun metagenomics enables the simultaneous and unbiased characterization of multiple biological components within a sample, including plant, bacterial, and viral DNA. This integrative capacity allows for a more comprehensive assessment of honey composition, as demonstrated here by the concurrent identification of botanical origin, microbial taxa, and viral content within the same dataset. However, several limitations inherent to this approach must be acknowledged. Shotgun metagenomics remains more costly and computationally demanding than metabarcoding, which may limit its widespread application in routine analyses. Additionally, the complexity of the honey matrix introduces challenges such as uneven DNA representation and potential dominance of host- or environment-derived DNA, which may influence detection sensitivity across taxa. As discussed above, factors such as low DNA input, absence of negative controls, and reliance on reference databases for taxonomic assignment further contribute to analytical uncertainty and highlight the importance of cautious interpretation.

Despite these limitations, the results of this pilot study suggest several relevant applications. In the context of honey authentication, shotgun metagenomics offers the potential to simultaneously verify botanical origin and detect inconsistencies that may indicate adulteration. From a food safety perspective, the ability to identify bacterial taxa, including potential pathogens, provides an additional layer of quality assessment, although the distinction between viable organisms and residual DNA must be carefully considered. Furthermore, the detection of bee-associated microorganisms and viruses, such as AmFV, supports the potential utility of this approach for non-invasive monitoring of bee health and colony-associated microbial dynamics.

Overall, while further validation in larger and systematically designed studies is required, shotgun metagenomics represents a promising and versatile tool for advancing the characterization of honey from both a biological and applied perspective.

The metagenomic profiles obtained from honey samples collected in Necochea, Buenos Aires province, Argentina, are broadly consistent with findings from previous shotgun metagenomic studies of honey, while also reflecting the distinctive agroecological characteristics of the Argentine Pampas region. The dominance of *Helianthus annuus* pollen reads in both Honey A (7.68% of total reads) and Honey B (5.69%) is in agreement with the prominent role of sunflower as a primary nectar and pollen source for AmFV colonies in Buenos Aires province, where the species is extensively cultivated as one of the most economically important oil crops in Argentina (Andrada et al., 2004). This botanical signature is consistent with the capacity of honey shotgun metagenomics to faithfully reflect the floral landscape surrounding the apiary, who showed that shotgun metagenomics profiles were concordant with melissopalynological descriptions of the botanical context of beehives (Galanis et al., 2022). The co-detection of *Eucalyptus grandis* reads in both samples further aligns with the regional apicultural landscape of Buenos Aires province, where eucalyptus plantations represent a key supplementary nectar source for honeybees (Fagúndez and Caccavari, 2006).

## 5 Conclusion

This pilot proof-of-concept study constitutes a first methodological and analytical step toward the comprehensive characterization of botanical and microbial diversity present in honey samples from Necochea, using an untargeted metagenomic approach, a strategy that remains novel and largely underexplored in the regional context for this biological matrix. Unlike traditional methods based on amplification of specific markers (such as ITS, rbcL, or 16S rRNA), the use of shotgun metagenomic sequencing combined with high-throughput taxonomic classification using bioinformatic tools such as Kraken2 enabled, in a single experiment, a multidimensional assessment of honey composition, encompassing floral origin as well as bacterial and viral communities associated with the apicultural environment.

The inclusion of a *R. chinensis* pollen control sample was essential for workflow validation, ensuring analytical specificity and sensitivity from DNA extraction through taxonomic assignment. Beyond confirming concordance with the expected taxonomic profile, the control sample provide a framework for interpreting background noise and the proportion of unassigned reads, a recurrent feature of metagenomics datasets likely attributable to incomplete reference databases and the intrinsic physicochemical complexity of the honey matrix.

Botanical profiling revealed a predominant contribution from *Helianthus annuus* (sunflower), followed by *Eucalyptus grandis*, consistent with the regional flora of Necochea. These findings highlight the potential of shotgun metagenomics as a robust alternative to traditional palynological analysis for botanical traceability, and suggest promising applications in denomination-if-origin studies, seasonal production monitoring, and quality certification frameworks.

At the microbial level, the detection of *Apilactobacillus kunkeei,* typical of fructose-rich environments and associated with hive health, together with soil bacteria such as *Bradyrhizobium* spp. and *Stutzerimonas stutzeri*, reflects the complex interaction among the plant communities, soil microbiota, and bee activity. The identification of *Paenibacillus larvae* in sample Honey A warrants particular attention, as this pathogen is responsible for American foulbrood and may have implications for colony surveillance and apicultural biosecurity. Conversely, the presence of *Acinetobacter species* and AmFV, commonly detected in environmental or endemic colony contexts, underscores the concept of honey as a biological record of the apicultural ecosystem, capturing both beneficial microorganisms as microbial symbiosis, and potentially pathogenic or stress-indicator taxa.

Although the proportion of unassigned reads (~60%) represents a current limitation shared with similar studies, this “metagenomic dark matter” it is also an opportunity: it underscores the need for expanded and curated genomic databases, especially for native plant species and apicultural microorganisms not yet sequenced. It also invites complementary multi-omic approaches (metatranscriptomics, metabolomics) to understand not only who is present, but what they are doing.

In conclusion, this work demonstrates that metagenomics is a powerful and versatile tool for the comprehensive analysis of honey, overcoming the limitations of targeted methods and providing a holistic view of its biological composition. The results obtained not only contribute to the scientific understanding of biodiversity associated with Argentine apiculture, but also lay the groundwork for future applications in quality control, traceability, colony health monitoring, and the development of value-added apicultural products. Future work should expand the sample cohort, incorporate seasonal sampling, and integrate physicochemical and sensory analysis of honey to develop predictive models linking the biological signatures with organoleptic attributes of honey.

## Data Availability

The raw sequencing data generated in this study are publicly available in the National Center for Biotechnology Information Sequence Read Archive (SRA) under BioProject accession PRJNA1458754.

## Footnotes

1
https://huttenhower.sph.harvard.edu/kneaddata/

2
https://huttenhower.sph.harvard.edu/kneadData_databases/Homo_sapiens_hg39_T2T_Bowtie2_v0.1.tar.gz
